# The Microbiota–Gut–Brain Axis in Insomnia: Mechanisms and Intervention Strategies

**DOI:** 10.3390/life16040583

**Published:** 2026-04-01

**Authors:** Mingze Yang, Qilin Chen, Zhizhou Meng, Xiaohong Gu, Chen Bai

**Affiliations:** 1 School of Traditional Chinese Medicine, Beijing University of Chinese Medicine, Beijing 100029, China; zymingze@163.com (M.Y.); tcchenqilin@163.com (Q.C.); mzz99108@163.com (Z.M.); 2 Institute of Chinese Medicine Epidemic Disease, Beijing University of Chinese Medicine, Beijing 102488, China

**Keywords:** microbiota–gut–brain axis, gut microbiota, microbial metabolites, insomnia, sleep disorders, sleep–wake cycle, probiotics, fecal microbiota transplantation

## Abstract

Insomnia is one of the most common sleep disorders. Traditionally, its pathophysiology has been interpreted mainly from the perspective of the central nervous system (CNS). However, accumulating evidence suggests that the microbiota–gut–brain axis (MGBA), a bidirectional communication network linking the gut and the CNS, may play an important role in the development, maintenance, and treatment of insomnia. This review summarizes the major signaling pathways of the MGBA and discusses its potential mechanisms in insomnia. Current evidence indicates that gut microbiota and their metabolites may influence sleep–wake homeostasis through neural, immune, endocrine, and circadian pathways. At the same time, insomnia-related stress responses, immune imbalance, and lifestyle disturbances may in turn affect the gut microbiota, thereby forming a bidirectional regulatory network. Animal and clinical studies further support a close association between gut microbial dysbiosis and insomnia. In addition, this review systematically summarizes factors that may affect the MGBA, including diet, lifestyle, psychosocial stress, medications, and medical exposures. On this basis, MGBA-targeted interventions, such as dietary modification, prebiotics and probiotics, lifestyle interventions, fecal microbiota transplantation, and natural medicines, may provide promising new strategies for the prevention and treatment of insomnia. Nevertheless, the current evidence still relies largely on animal studies and cross-sectional research, and further longitudinal studies and high-quality interventional trials are needed to clarify causality, long-term efficacy, and standardized therapeutic approaches.

## 1. Introduction

With accelerating urbanization, increasing psychological stress, and changes in lifestyle, sleep disorders have become a major public health concern [1]. A recent global analysis based on a systematic review of the literature estimated that approximately 16.2% of adults worldwide, corresponding to about 852 million individuals, suffer from insomnia [2]. Women [2], older adults [3], and individuals with greater stress reactivity [4] appear to be particularly vulnerable. It should be noted, however, that the reported prevalence of insomnia is substantially influenced by the diagnostic criteria and assessment methods used, which contributes to marked variation across studies and regions [5]. For example, prevalence estimates in the same population may differ considerably depending on the diagnostic standard applied, ranging from 8.5% under the Diagnostic and Statistical Manual of Mental Disorders, Fifth Edition (DSM-5) criteria to 20.0% under the International Classification of Sleep Disorders, Third Edition (ICSD-3) criteria [6].

Within the traditional neurobiological framework, hyperarousal is widely regarded as a core mechanism underlying insomnia [7]. In this context, multiple processes, including neurotransmitter imbalance [8], enhanced activation of stress-related neuroendocrine systems [7], immune–inflammatory activation [9], circadian rhythm disruption [10], and cognitive–emotional dysregulation [11], may contribute to the onset and progression of insomnia and jointly promote or maintain a state of hyperarousal. Chronic insomnia not only impairs sleep quality and daytime functioning but is also closely associated with a range of adverse health outcomes. Increasing evidence indicates that insomnia may interact bidirectionally with psychiatric conditions such as anxiety and depression [12,13]. In addition, insomnia has been significantly associated with metabolic and cardiovascular diseases [14,15]. These findings suggest that traditional hypotheses centered exclusively on the central nervous system (CNS) are insufficient to fully explain the multisystem and multidimensional nature of insomnia. This has prompted increasing interest in peripheral regulatory systems, particularly the gut microbiota (GM) and its bidirectional communication network with the CNS.

The microbiota–gut–brain axis (MGBA) is a complex bidirectional communication system linking the gastrointestinal tract and the CNS, primarily through neural, endocrine, and immune pathways [16]. As a key component of this axis, the GM plays an essential role in maintaining host physiological homeostasis and modulating brain function through multiple mechanisms. For instance, the GM produces metabolites such as short-chain fatty acids (SCFAs), which participate in the synthesis and regulation of neurotransmitters. In addition, the GM may influence CNS function by activating vagal afferent signaling and modulating immune–inflammatory and neuroendocrine responses [16,17].

In recent years, accumulating evidence has shown that, compared with healthy individuals, patients with insomnia exhibit significant alterations in gut microbial composition and diversity. These changes may include reduced alpha diversity, altered beta diversity, decreased relative abundance of beneficial taxa such as *Faecalibacterium*, and increased abundance of opportunistic or potentially pathogenic taxa, including members of Actinomycetota and *Bacteroides* [18]. Such dysbiosis may disrupt sleep regulation by affecting neurotransmitter homeostasis, promoting inflammatory signaling cascades, and enhancing the reactivity of the hypothalamic–pituitary–adrenal (HPA) axis [16,17].

In this review, we summarize the major signaling pathways of the MGBA, its potential mechanistic links to insomnia, and the available clinical and experimental evidence. We further discuss factors that may influence sleep through the MGBA and outline emerging microbiota-targeted intervention strategies, with the aim of providing an expanded framework for understanding the pathophysiology of insomnia and identifying potential therapeutic opportunities.

## 2. The MGBA and Its Bidirectional Signaling Pathways

### 2.1. Composition and Functions of the GM

The GM is a complex microbial community that colonizes the human gastrointestinal tract and includes bacteria, fungi, viruses, and archaea [19]. The GM not only participates in food digestion and nutrient transformation, but also helps maintain intestinal barrier integrity by regulating intestinal epithelial cells, mucus secretion, and the expression of tight junction proteins [20]. In addition, through its interactions with the host immune system, the GM contributes to the establishment of immune tolerance and the regulation of inflammatory responses, thereby playing an essential role in maintaining immune homeostasis and metabolic balance [21,22].

In general, greater microbial diversity and a more stable community structure are considered to reflect a healthier intestinal ecosystem [23], whereas dysbiosis has been implicated in the development and progression of a wide range of diseases. For example, patients with obesity and diabetes often exhibit alterations in microbiota related to energy metabolism, including a reduced abundance of SCFA-producing bacteria and an increase in pro-inflammatory taxa [24,25]. Systematic reviews and meta-analyses have also shown that patients with rheumatic and immune-related diseases display marked gut microbial disturbances, which are associated with immune dysregulation and increased exposure to self-antigens [26]. In the field of neuropsychiatry, patients with depression and anxiety disorders frequently show abnormalities in gut microbial composition, including reduced microbial diversity and altered abundance of specific taxa [27]. Increasing evidence suggests that the GM is not only a regulator of peripheral metabolic and immune homeostasis, but may also participate in the regulation of CNS function through neural, immune, and endocrine pathways [28].

### 2.2. Neural Pathways

Neural signaling is one of the fastest and most direct modes of communication within the MGBA. This bidirectional communication is mediated primarily by the autonomic nervous system (ANS) and enables continuous information exchange between the gut and the CNS. Sensory signals originating in the gut are transmitted to the CNS through vagal and spinal afferent pathways, whereas the CNS regulates intestinal motility, secretion, and barrier function through sympathetic and parasympathetic efferent outputs [29]. Together, these neural circuits provide the structural basis for gut–brain communication, allowing microbial-derived metabolic signals to be integrated with neural regulatory networks and thereby influence brain function and behavior.

#### 2.2.1. Vagal Pathway

The vagus nerve is the principal neural pathway connecting the enteric nervous system (ENS) and the CNS, and it plays a dominant role in gut–brain communication. Mechanical stimuli, nutrients, chemical signals, and microbial metabolites within the intestinal lumen are sensed and integrated by the ENS and enteroendocrine cells (EECs) and are subsequently transmitted via vagal afferents to the nucleus tractus solitarius (NTS) in the brainstem [30,31]. Vagus-associated gut–brain signaling can further influence neural activity in limbic and reward-related brain regions [32]. In addition, this pathway may regulate hippocampus-related memory processes and neural plasticity, thereby affecting specific cognitive functions [33].

A variety of microbial metabolites can influence vagal activity through receptor-mediated mechanisms. For example, SCFAs may modulate the activity of vagal afferent neurons through pathways involving free fatty acid receptor 3 (FFAR3) [34]. Bile acids may also regulate vagal signaling through G protein-coupled bile acid receptor 5 (TGR5)-mediated mechanisms, thereby enhancing signal transmission from the gut to the brainstem [35]. In addition, enterochromaffin cells in the gut synthesize large amounts of peripheral 5-hydroxytryptamine (5-HT), which may act on specific 5-HT receptor subtypes expressed on vagal afferent fibers, thereby modulating central monoaminergic systems and influencing emotional and stress-related responses [36].

#### 2.2.2. ENS

The ENS is often referred to as the “second brain” and represents the largest division of the peripheral nervous system. It consists of many neurons and glial cells and shares substantial structural and functional similarities with the CNS. The ENS expresses many of the same neurotransmitters, receptors, and transcription factors as the CNS, forming complex local neural circuits [37].

First, the ENS can directly sense changes in the intestinal microenvironment and regulate multiple physiological activities, including gut motility, secretion, and blood flow, through local reflex circuits [38,39]. At the same time, it integrates signals from GM, EECs, and immune cells, and modulates neurotransmitter release and local neural activity, thereby contributing to intestinal homeostasis [40]. Previous studies have shown that ENS dysfunction is associated with a variety of neurological and neurodevelopmental disorders, including Alzheimer’s disease, Parkinson’s disease, and autism spectrum disorder [37]. These findings suggest that structural and functional alterations in the ENS may affect the transmission of gut-derived signals to central neural circuits.

#### 2.2.3. Spinal Afferent Pathway

Spinal afferent nerves constitute another important route by which gut-derived sensory information is transmitted to the CNS. The cell bodies of these neurons are located in the dorsal root ganglia (DRG), and their peripheral terminals are distributed within the intestinal wall, where they can detect a variety of signals, including mechanical distension, chemical mediators, inflammation, and noxious stimuli [41]. These signals are first processed in the superficial layers of the dorsal horn of the spinal cord, where they are integrated with visceral and nociceptive inputs under the combined regulation of local excitatory and inhibitory circuits [42]. Subsequently, the relevant signals are conveyed to the brainstem, thalamus, and limbic system through ascending pathways such as the spinothalamic and spin reticular tracts, thereby participating in central sensory regulation [42]. In addition, descending pathways originating from the periaqueductal gray and the rostral ventromedial medulla modulate dorsal horn excitability, forming a bidirectional regulatory network [43].

Unlike the vagus nerve, which mainly transmits homeostatic and metabolic signals, spinal afferents are particularly sensitive to inflammatory mediators and tissue injury. Pro-inflammatory cytokines, bacterial endotoxins, and gut microbial metabolites may alter the excitability of DRG neurons, thereby influencing central neural processing and neuroimmune interactions [44].

### 2.3. Endocrine Pathways

Endocrine signaling represents another major component of the MGBA and primarily mediates gut–brain communication through circulating hormones and neuroactive peptides. EECs, which are distributed throughout the intestinal epithelium, are increasingly recognized as key gatekeepers of the MGBA [45]. These cells can sense luminal nutrients and microbial metabolites and release a variety of bioactive molecules, including 5-HT, cholecystokinin (CCK), glucagon-like peptide-1 (GLP-1), and peptide YY (PYY). These signaling molecules may further influence central regulatory circuits through humoral or neural pathways [31], thereby linking gut-derived signals with host metabolic and neuroregulatory processes.

Among these mediators, 5-HT functions as both a neurotransmitter and a hormone. Approximately 90% of the body’s 5-HT is synthesized by enterochromaffin cells from dietary tryptophan. Peripheral 5-HT may indirectly modulate central neural networks through vagal afferent signaling, immune regulation, and metabolic pathways [46]. In addition to 5-HT, peptide hormones secreted by EECs also play important roles in the MGBA. GLP-1, CCK, and PYY can act on receptors in the brainstem and hypothalamus and are involved in the regulation of feeding behavior, emotional state, and energy metabolism [31,47,48]. Among these peptides, GLP-1 has attracted particular attention in recent years. Studies have shown that GLP-1 can activate intracellular signaling pathways such as PI3K/Akt, promote neuronal survival, and attenuate oxidative stress and neuroinflammation. Moreover, GLP-1 receptor agonists have been shown to improve neuronal dysfunction and reduce inflammatory injury in several models of neurodegenerative disease [47,49].

The HPA axis represents another key neuroendocrine pathway linking the gut and the brain and serves as a major effector system in the stress response. GM and their metabolites may jointly regulate HPA axis activity through immune–inflammatory signaling and vagal afferent input, thereby influencing the corticotropin-releasing hormone–adrenocorticotropic hormone–cortisol cascade [50]. Sustained elevation of cortisol may induce structural and functional alterations in the hippocampus, prefrontal cortex, and other emotion-related brain regions, leading to reduced neuroplasticity and decreased expression of neurotrophic factors. These changes are considered to be closely related to the development and progression of psychiatric disorders [51].

At the same time, activation of the HPA axis not only affects the CNS, but also feeds back on intestinal physiology. Elevated cortisol levels may impair intestinal barrier integrity, increase intestinal permeability, and promote mucosal immune activation and inflammatory responses, thereby exacerbating gut dysbiosis and systemic low-grade inflammation [52]. Animal studies have further shown that chronic glucocorticoid exposure can induce anxiety-like and depression-like behaviors and is accompanied by alterations in gut microbial composition and brain metabolic profiles [53].

### 2.4. Immune–Inflammatory Pathways

The gut contains the largest proportion of immune cells in the body [54] and can sense changes in the GM and their metabolites. By regulating innate and adaptive immune responses, the release of inflammatory mediators, and barrier function, the gut immune system can influence both peripheral and central inflammatory states [55]. At the same time, the CNS can regulate intestinal immune responses through neural and endocrine mechanisms, thereby forming a bidirectional immune–inflammatory regulatory pathway within the MGBA [56].

When gut dysbiosis occurs or intestinal barrier function is impaired, intestinal immune homeostasis may be disrupted, leading to increased levels of pro-inflammatory cytokines and endotoxins and promoting chronic low-grade inflammation [57]. These inflammatory mediators, including interleukin (IL)-1β, IL-6, and tumor necrosis factor (TNF)-α, may disrupt the blood–brain barrier (BBB) [58], activate microglia, and alter neurotransmitter homeostasis and synaptic remodeling through cytokine receptor-related signaling pathways, ultimately affecting CNS function [59].

In addition to humoral inflammatory signaling, cytokines released by intestinal immune cells may directly activate vagal afferent terminals and transmit signals to the NTS in the brainstem, thereby influencing higher-order neural circuits [60]. Meanwhile, the CNS can regulate peripheral immune responses through the ANS. One of the key mechanisms is the cholinergic anti-inflammatory pathway mediated by vagal signaling, in which acetylcholine suppresses the pro-inflammatory responses of immune cells such as macrophages [61]. In contrast, persistent sympathetic activation under chronic stress tends to amplify inflammatory responses and impair intestinal epithelial barrier function [62].

Furthermore, the HPA axis is an important upstream regulator of immune–inflammatory responses. In the short term, stress-induced glucocorticoid elevation generally exerts immunosuppressive and anti-inflammatory effects [63]. However, chronic or repeated overactivation of the HPA axis may disrupt immune homeostasis and further amplify intestinal and central inflammatory responses by affecting the intestinal barrier, microbial composition, and inflammatory milieu [62,63].

**Figure 1:**
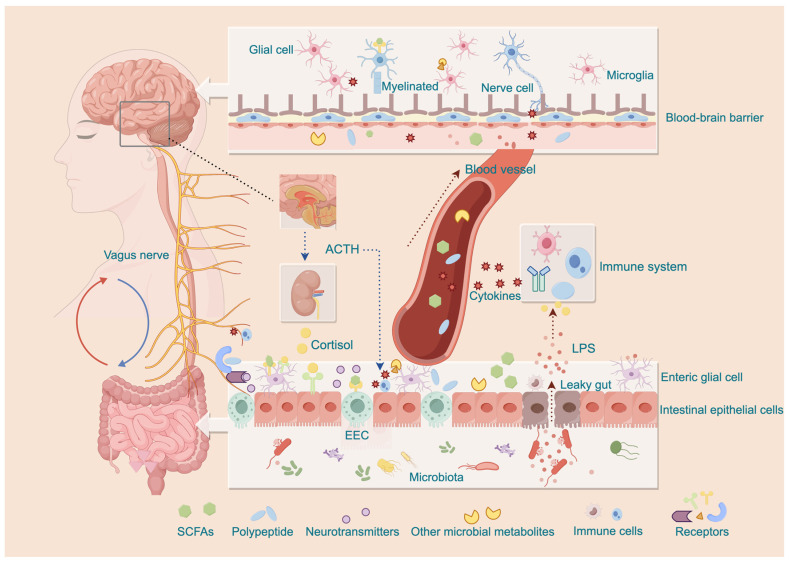
Bidirectional signaling pathways of the microbiota–gut–brain axis (red arrows indicate signaling from the gut to brain, whereas blue arrows indicate signaling from the brain to gut). The central nervous system regulates intestinal function via the vagus nerve and the hypothalamic–pituitary–adrenal axis, including release of adrenocorticotropic hormone (ACTH) and cortisol (blue dashed arrows), thereby modulating gut permeability, barrier integrity, and immune activity. Conversely, gut-derived signals reach the brain through several interconnected routes: (i) neural pathways, including vagal and spinal afferents activated by microbial metabolites (e.g., short-chain fatty acids (SCFAs)), enteroendocrine cell (EEC)-derived neurotransmitters (e.g., 5-hydroxytryptamine) and cytokines released from immune cells; (ii) immune–inflammatory pathways, in which disruption of the intestinal barrier allows lipopolysaccharide (LPS) to activate immune cells and induce cytokine production (right red dashed arrows), with further mediation through local crosstalk between immune cells and neurons; (iii) neuroendocrine pathways, through which gut hormones and other circulating mediators influence central signaling (central red dashed arrows).

Taken together, the MGBA constitutes a highly interconnected and dynamically regulated bidirectional communication network through the coordinated actions of neural, endocrine, and immune–inflammatory pathways, thereby contributing to the maintenance of homeostasis between the gut and the CNS (Figure 1).

## 3. Mechanistic Links Between the MGBA and Insomnia

Sleep initiation and maintenance are complex physiological processes. Within the traditional neurobiological framework, insomnia is generally considered to result from dysregulation of the sleep–wake regulatory system [64]. Among the proposed mechanisms, hyperarousal is regarded as one of the core pathophysiological features of insomnia, reflecting an imbalance between wake-promoting and sleep-promoting systems [7]. In addition, neurotransmitter abnormalities, immune–inflammatory activation, stress-related neuroendocrine dysregulation, and circadian rhythm disturbance are all thought to contribute to the onset and chronicity of insomnia [8,64,65].

In recent years, accumulating evidence has suggested that gut microbial dysbiosis and related abnormalities of the MGBA may also serve as important peripheral regulators of sleep–wake homeostasis [18,66]. By linking the GM, ENS, immune system, and CNS, the MGBA forms a multidimensional and bidirectional regulatory network. Through its potential effects on neurotransmitter metabolism, stress responsivity, immune–inflammatory status, and circadian rhythms, the MGBA may participate in the development and progression of insomnia [66].

### 3.1. MGBA and Neurotransmitter Imbalance

Unlike the traditional view, which focuses primarily on central neurotransmitters, the MGBA emphasizes that the gut is not only an organ of nutrient absorption, but also an important site for the generation, transformation, and integration of multiple neuroactive molecules. The contribution of the MGBA to insomnia-related neurotransmitter imbalance may be reflected in two main aspects. On the one hand, it may influence central neurotransmitter homeostasis by regulating tryptophan metabolism, SCFAs, and other microbiota-derived metabolites. On the other hand, although gut-derived neuroactive molecules do not readily cross the BBB, they may indirectly modulate the function of sleep-related brain regions through communication pathways involving the vagus nerve, immune–inflammatory signaling, and neuroendocrine regulation.

First, the MGBA may participate in central neurotransmitter homeostasis by affecting neurotransmitter precursors and related metabolic pathways. Tryptophan is a key precursor of 5-HT, and its intestinal metabolism is jointly regulated by the host and the GM, mainly through the 5-HT, kynurenine, and indole pathways [67]. Under conditions of dysbiosis, tryptophan metabolism may shift toward the kynurenine pathway, resulting in reduced 5-HT availability and potentially impaired sleep regulation [67]. Among the metabolites of this pathway, elevated kynurenic acid (KYNA) has been associated with reduced rapid eye movement (REM) sleep, decreased delta power during non-rapid eye movement (NREM) sleep, and fewer sleep spindles, whereas inhibition of KYNA synthesis has been shown to reverse these abnormalities [68]. The indole derivative indole-3-acetic acid (IAA) may also participate in sleep regulation. Previous studies have shown that IAA can promote 5-HT release by activating the aryl hydrocarbon receptor in enterochromaffin cells and may alleviate cognitive deficits associated with sleep deprivation [69].

In addition, tyrosine-related signals generated through microbial fermentation may have the potential to enter brain tissue [70], and tyrosine itself is an important precursor of catecholamine neurotransmitters. Among sleep-related neurotransmitters, gamma-aminobutyric acid (GABA) is a major inhibitory neurotransmitter in the CNS. It is closely associated with sleep initiation and maintenance and may reduce neuronal excitability, shorten sleep latency, prolong NREM sleep, and improve sleep quality [8,71].

Second, enterochromaffin cells, the ENS, and the GM jointly participate in the production and regulation of gut-derived neurotransmitters such as 5-HT, GABA, and dopamine. It has been proposed that gut-derived 5-HT and GABA may act on receptors located on vagal afferent terminals, transmit signals to the NTS, and further influence sleep-related brain regions, including the dorsal raphe nucleus, locus coeruleus, and hypothalamus, thereby regulating sleep stability and arousal levels [36,72]. In addition, peripheral dopaminergic signals, including microbiota-derived dopamine-related metabolites, may influence central dopamine availability and signaling through vagal, immune–inflammatory, and HPA axis-related mechanisms, thereby potentially contributing to sleep–wake regulation [73].

### 3.2. MGBA and HPA Axis Dysregulation

Dysregulation of the HPA axis is considered one of the major neuroendocrine bases of insomnia. Abnormal activation of this axis is associated with sustained cortisol elevation and a state of hyperarousal, thereby contributing to the onset and maintenance of insomnia [74]. According to the 3P model of insomnia, stressful life events, psychological burden, and environmental disturbances often act as precipitating factors, whereas pre-existing traits such as high sleep reactivity constitute a host vulnerability. When these factors converge, insomnia is more likely to be triggered and maintained [4,75].

Recent studies further suggest that oscillations in the GM may regulate the diurnal rhythm of the HPA axis. Microbiota depletion has been shown to disrupt stress-related transcriptional and metabolic profiles in the hippocampus and amygdala and to induce abnormalities in glucocorticoid rhythmicity, whereas fecal microbiota transplantation (FMT) may partially restore these changes [76]. Under stress conditions, elevated glucocorticoid levels may alter intestinal permeability and microbial composition, resulting in a reduction in beneficial bacteria and an expansion of potentially pathogenic taxa. These changes may further affect sleep- and emotion-related neural regulatory processes through the gut–brain axis [52,77]. In this context, insomnia may arise from the interaction between stress exposure and host susceptibility, with gut dysbiosis serving as an important mediating factor.

Among microbiota-related metabolites, SCFAs may influence HPA axis activity through epigenetic regulation and neuroendocrine pathways, thereby modulating the stress response and potentially alleviating stress-related hyperarousal [78]. Gut-derived GABA may also reduce stress by inhibiting HPA axis activity and may thereby contribute to physiological processes related to emotion and sleep [79]. In addition, hormones such as GLP-1, CCK, and PYY may influence hypothalamic signaling through humoral or vagal pathways, participate in the regulation of energy metabolism and feeding behavior, and potentially affect the sleep–wake state [80].

Importantly, insomnia and the accompanying HPA axis hyperactivity may also feedback on the GM, thereby forming a vicious cycle. Animal studies have shown that sleep deprivation is accompanied by HPA axis activation and elevated corticosterone levels, as well as marked alterations in gut microbial composition, such as a reduced abundance of *Lactobacillus*, together with depletion of beneficial metabolites including SCFAs. These changes may further aggravate sleep disturbance [81].

### 3.3. MGBA and Immune–Inflammatory Abnormalities

Immune–inflammatory dysregulation is regarded as one of the major pathological bases of insomnia and represents a key link between gut microbial alterations and CNS function. Increasing evidence indicates a clear bidirectional relationship between sleep and inflammation. On the one hand, peripheral and central inflammatory processes can disrupt sleep architecture and sleep homeostasis [82]. On the other hand, persistent insomnia itself may induce immune imbalance and amplify inflammatory responses, thereby creating a vicious cycle [83]. Notably, sustained inflammatory activation may also interact with the HPA axis and circadian regulatory systems, and may be accompanied by disrupted diurnal cortisol secretion and enhanced physiological arousal [84].

Clinical studies provide support for this association. Patients with insomnia have been reported to exhibit elevated levels of inflammatory markers, including C-reactive protein (CRP), IL-6, and TNF-α [83]. These inflammatory mediators may not only affect neuronal function and synaptic plasticity, but may also contribute to abnormal sleep regulation by disturbing neurotransmitter systems involved in sleep [83]. In particular, IL-1β and TNF-α are closely associated with NREM sleep and slow-wave activity and may influence sleep drive [85]. In addition, other inflammation-related signaling molecules, such as adenosine, chemokines, and reactive oxygen species, may also affect sleep architecture and sleep homeostasis [86].

Within the MGBA, impairment of the intestinal mucosal barrier is considered an important initiating event in peripheral inflammation. Gut dysbiosis may weaken the integrity of the intestinal epithelial barrier, increase intestinal permeability, and facilitate the entry of microbiota-related products into the circulation, thereby triggering peripheral immune responses and the release of pro-inflammatory cytokines [20]. These inflammatory signals may further act on the BBB, increase its permeability, and promote the transmission of peripheral inflammatory signals to the CNS [58]. Among them, IL-1β and TNF-α may directly affect central sleep-regulatory networks and related neurotransmitter systems, thereby altering sleep structure and reducing sleep quality [86].

Gut microbial metabolites also play important immunomodulatory roles in this process. As major microbiota-derived metabolites, SCFAs may help maintain immune homeostasis through G protein-coupled receptor signaling and inhibition of histone deacetylases (HDACs), thereby potentially attenuating the disruptive effects of inflammation on central sleep-regulatory circuits [87]. Gut-derived 5-HT may act on immune cells such as dendritic cells, macrophages, and T cells through specific 5-HT receptors, thereby influencing inflammatory mediator release and the intestinal microenvironment [88]. Gut-derived GABA may also modulate HPA axis activity through peripheral signaling and hypothalamic integration, reduce stress-related neuroendocrine responses, and indirectly participate in sleep-related network regulation by improving the intestinal microenvironment and modulating peripheral inflammatory status [72].

In addition, although direct evidence linking the tryptophan metabolites 3-hydroxykynurenine (3-HK) and quinolinic acid (QUIN) to insomnia remains limited, their inflammation-related and neurotoxic biological effects suggest a potential role in sleep disturbance. Under inflammatory conditions, 3-HK may promote reactive oxygen species generation and microglial activation, thereby disrupting neural homeostasis and potentially contributing to the development of sleep disorders [89]. QUIN, a potent N-methyl-D-aspartate receptor agonist, may induce calcium influx, oxidative stress, and neuronal injury, thereby increasing arousal and contributing to sleep disruption [90].

### 3.4. MGBA and Circadian Rhythm Disturbance

Circadian rhythm disruption is another core feature of insomnia. Increasing evidence suggests that the MGBA exerts bidirectional effects on host circadian regulation. On the one hand, host core clock genes may shape the diurnal oscillatory patterns of the GM by regulating feeding–fasting rhythms, local intestinal epithelial function, and the immune microenvironment [91]. Disruption of intestinal epithelial clock function, such as *Bmal1* deficiency, has been shown to markedly attenuate microbial rhythmicity [92].

On the other hand, microbiota-derived metabolites, especially SCFAs, may in turn regulate the host peripheral clock system [93]. SCFAs, particularly butyrate and propionate, have epigenetic regulatory properties and may influence the expression of circadian genes such as *PER2* through inhibition of HDACs, suggesting a potential role in the maintenance of circadian homeostasis [94]. In addition, bile acids, as host–microbiota co-metabolites, exhibit pronounced circadian rhythmicity in their synthesis and transport, and sleep deprivation may disrupt these rhythms [95]. Gut-derived hormone secretion, such as GLP-1, also shows circadian fluctuation, and sleep deprivation or circadian disruption may alter its secretory rhythm, suggesting its possible involvement in the metabolic regulation related to the sleep–wake cycle [96].

Melatonin, a key hormone involved in circadian regulation and sleep initiation, may also be influenced by the GM. Previous studies suggest that the GM may indirectly promote melatonin synthesis and secretion by modulating SCFAs and tryptophan-derived metabolites [97]. In turn, melatonin may also regulate microbial composition and function, thereby forming a reciprocal regulatory loop [98].

### 3.5. Experimental and Clinical Evidence Supporting an Association Between GM and Insomnia

#### 3.5.1. Evidence from Animal Models: Causality

Animal studies provide relatively strong evidence supporting a causal role of the GM in sleep regulation. First, in microbiota-depletion models, antibiotic treatment or germ-free status has been shown to alter both sleep–wake architecture and electroencephalographic power spectra in mice. One study reported that, after 4 weeks of broad-spectrum antibiotic treatment, amino acid and vitamin metabolism related to neurotransmitter function was significantly altered in murine cecal contents, with decreased levels of metabolites such as 5-HT and vitamin B6 [99]. At the same time, sleep analysis revealed abnormal distribution of NREM sleep, altered REM-related rhythms, and instability in sleep–wake transitions, suggesting that the GM contributes to the maintenance of normal sleep architecture [99].

Second, FMT has further strengthened causal inference. Transplantation of microbiota from patients with insomnia into germ-free mice induced insomnia-like phenotypes in the recipient animals, including frequent awakenings and shortened NREM duration, together with reduced serum butyrate levels [100]. In addition, transplantation of fecal microbiota from sleep-deprived donors into healthy recipients was able to transfer certain adverse phenotypes, including depression-like behavior, impaired cardiac function, and myocardial fibrosis [101]. These findings suggest that the GM may mediate systemic pathological consequences associated with sleep disturbance.

#### 3.5.2. Clinical Observational Evidence: Association

Clinical studies likewise support an association between GM and insomnia. Compared with healthy controls, patients with insomnia often exhibit alterations in gut microbial structure and diversity. Some studies have reported reduced alpha diversity, together with depletion of specific beneficial taxa and relative enrichment of potentially pathogenic microorganisms (Table 1). Although the specific taxa reported to differ are not entirely consistent across studies, the overall trend supports an association between insomnia and gut dysbiosis.

In addition to structural alterations in the microbiota, patients with insomnia often show abnormalities in microbial metabolic function. A case–control study found that total fecal SCFAs, including acetate, propionate, butyrate, and valerate, were significantly lower in patients with insomnia than in healthy controls, and that SCFA levels were significantly negatively correlated with both the Pittsburgh Sleep Quality Index (PSQI) and the Insomnia Severity Index (ISI) [110]. Another study in patients with sleep disorders found significant enrichment of the “phenylalanine, tyrosine, and tryptophan biosynthesis” pathway among gut metabolites, suggesting dysregulation of neurotransmitter precursor-related metabolism [111]. In addition, other microbiota-related metabolites also showed characteristic alterations. Eleven serum metabolites, including adenosine, phenol, and phenyl sulfate, were significantly altered in patients with insomnia [112]. Further correlation analyses showed that adenosine levels were positively correlated with the abundance of *Lachnospira* and with total sleep time, whereas phenol and phenyl sulfate levels were negatively correlated with the abundance of *Coprococcus* and positively correlated with PSQI and ISI scores [112]. Collectively, these findings suggest that abnormalities in microbiota-related metabolites may represent an important link between gut dysbiosis and the phenotypic manifestations of sleep disturbance.

## 4. Factors Influencing the MGBA in Insomnia

Sleep regulation is influenced by both exogenous and endogenous factors, many of which may exert their effects through modulation of the MGBA. Exogenous factors include diet, lifestyle, medication exposure, and circadian disruption related to environmental factors, all of which may alter microbial composition and metabolic output [113,114,115,116]. Endogenous factors, such as host neuroendocrine responsivity, immune status, genetic susceptibility, and intrinsic circadian mechanisms, may further regulate microbial dynamics and host–microbiota signaling [117]. Through bidirectional interactions, these factors may influence sleep homeostasis and circadian stability, thereby contributing to the development and persistence of insomnia.

### 4.1. Diet and Lifestyle

Diet exerts a profound impact on the composition and metabolic function of the GM. Unhealthy dietary patterns, such as high-fat and high-sugar intake, can disrupt gut microbial homeostasis [118,119]. High-fat intake may promote the expansion of pro-inflammatory microbial taxa, impair intestinal barrier integrity, and trigger immune activation and endogenous stress responses, which may be unfavorable for the maintenance of sleep homeostasis [118,120]. Excessive sugar intake may lead to glycemic instability and has been associated with increased nocturnal awakenings and sleep fragmentation [121]. In addition to macronutrients, exposure to ultra-processed food components, such as artificial sweeteners and emulsifiers, may reshape microbial ecology and barrier function [122], suggesting a potential role in sleep-related MGBA signaling, although insomnia-specific evidence remains limited.

Lifestyle factors may also modulate both the GM and sleep. Regular moderate physical activity is generally associated with a healthier microbial profile and better sleep quality [123], whereas sedentary behavior may contribute to alterations in microbial composition and function [124] and has been associated with an increased risk of insomnia [125]. Although short-term alcohol intake may exert sedative effects, repeated alcohol consumption before sleep can disrupt sleep architecture, increase nocturnal awakenings, and reduce overall sleep quality [126]. Alcohol may also increase intestinal permeability and inflammatory signaling, alter microbial composition, and indirectly affect emotion, cognition, and sleep-related behaviors [127,128]. A cross-sectional study showed that individuals who adhered to multiple healthy behaviors, including a balanced diet, regular exercise, non-smoking, limited alcohol use, reduced sedentary time, and maintenance of normal body weight, had better sleep quality, more appropriate sleep duration, and a lower risk of insomnia [129]. These findings highlight the importance of exogenous factors in the regulation of the MGBA.

Environmental exposure is another factor that should not be overlooked. Emerging evidence suggests that fine particulate matter, such as PM2.5, may impair intestinal barrier integrity and promote inflammatory activation, thereby potentially affecting MGBA pathways related to sleep stability [130]. In addition, circadian disruption, including shift work and nighttime exposure to artificial light, may lead to desynchronization between microbial diurnal oscillations and host circadian rhythms, thereby disturbing sleep regulation [131].

### 4.2. Stress and Psychological Factors

Stress is an important psychosocial factor influencing the MGBA. Psychosocial stressors such as occupational strain, interpersonal conflict, and major life events may activate the sympathetic nervous system and disrupt the HPA axis, thereby impairing sleep regulation [132,133]. Psychological disorders such as anxiety and depression commonly co-occur with insomnia and may interact with insomnia in a bidirectional manner [12,134]. Negative emotions, maladaptive cognition, and chronic perceived stress may not only directly impair sleep quality [135,136,137], but may also be accompanied by gut microbial imbalance, thereby contributing to the interaction between psychological factors and sleep disturbance. Studies have shown that patients with anxiety and depression often exhibit abnormalities in gut microbial composition, with some reports indicating reduced microbial diversity and decreased abundance of beneficial taxa [27,138]. These findings suggest that the GM may participate in the association between psychological factors and sleep. Clinical evidence further shows that cognitive behavioral therapy can improve insomnia symptoms and exert beneficial effects on comorbid anxiety and depressive symptoms [139,140], highlighting the importance of addressing psychological factors in the comprehensive management of insomnia.

### 4.3. Drug- and Medical-Related Factors

Drug and medical exposures represent another category of exogenous factors that may affect the stability of the MGBA. Antibiotics are among the most important disruptors of the GM, and their use can markedly alter microbial composition, diversity, and metabolic capacity, leading to dysbiosis as well as disturbances in immune and neurochemical signaling [141,142]. These effects appear to be particularly pronounced in neonates and children and may have long-term consequences [143].

Animal experiments have shown that antibiotic-induced microbiota depletion can disrupt neurotransmitter-related metabolism and alter sleep architecture [99]. Long-term exposure may also be accompanied by changes in metabolic pathways and neurobehavioral abnormalities, including anxiety-like behavior [144].

In addition to antibiotics, hormonal agents, such as estrogens, progestins, and thyroid hormones, may affect microbial growth, adhesion, biofilm formation, and SCFA profiles [145]. Glucocorticoids, such as dexamethasone, may reduce microbial abundance and diversity and are associated with immunosuppression and metabolic alterations [146], potentially influencing sleep by disrupting the feedback loops among the HPA axis, immunity, and the GM. Analgesics, particularly opioids, may also induce dysbiosis and affect behavioral regulation; meanwhile, pain treatment itself is closely associated with alterations in sleep architecture [147,148].

Clinical observations suggest that cancer treatment-related neuropsychiatric symptoms may be associated with abnormalities in the GM, including sleep-related symptoms, indicating that dysbiosis induced by chemotherapy, radiotherapy, and other medical exposures may contribute to sleep disturbance [149]. Surgery and anesthesia may likewise induce gut microbial alterations and interfere with tryptophan–kynurenine metabolism, leading to impaired barrier function and postoperative sleep disturbance [150].

## 5. MGBA-Targeted Therapeutic Strategies for Insomnia

Given that the GM may participate in the development and maintenance of insomnia through pathways involving neurotransmitter metabolism, HPA axis function, immune–inflammatory regulation, and circadian rhythms, interventions targeting the MGBA have emerged as a promising direction for insomnia treatment (Table 2).

### 5.1. Dietary Approaches

Dietary modification represents a fundamental and readily implementable strategy for regulating the MGBA. Observational studies have shown that adherence to a Mediterranean diet, characterized by high intake of vegetables, whole grains, legumes, nuts, and olive oil, is associated with improved sleep quality and reduced insomnia severity [164,165]. In addition, regular daytime-centered eating patterns, such as early time-restricted feeding, may help maintain synchrony between the GM and host circadian rhythms. In contrast, nighttime eating or irregular meal timing may contribute to circadian misalignment, disrupt microbial diurnal oscillations, and promote dysbiosis, thereby potentially increasing the risk of insomnia [166,167].

Beyond overall dietary patterns, specific nutrients have also been associated with sleep quality. A review has suggested that high-carbohydrate diets and foods rich in tryptophan, melatonin, and phytonutrients, such as cherries, are associated with improved sleep outcomes, potentially through mechanisms involving modulation of 5-HT and melatonin [168]. Dietary polyphenols, including catechins, anthocyanins, and resveratrol, may modulate gut microbial composition and reduce oxidative stress and inflammatory responses, thereby conferring potential benefits for sleep quality [169,170]. In addition, high-fiber diets may increase the abundance of beneficial bacteria and promote SCFA production, which may help maintain intestinal mucosal barrier integrity, attenuate inflammation, and preserve metabolic signaling homeostasis within the gut-brain axis [171,172].

### 5.2. Prebiotics and Probiotics

Prebiotics, as selectively fermentable substrates, can promote the growth of beneficial microorganisms and thereby improve the intestinal microenvironment [173]. Animal studies have shown that prebiotic-enriched diets can alter fecal microbial composition and promote sleep recovery following sleep deprivation [174]. In models of chronic circadian disruption, prebiotic intervention has also been shown to modulate microbial composition and bile acid profiles, facilitate sleep/circadian resynchronization, and potentially alleviate comorbid anxiety and depressive symptoms [154].

At present, probiotics are receiving increasing attention as a non-pharmacological approach for sleep disorders. Several randomized controlled trials have shown that supplementation with specific probiotic strains may improve sleep quality or extend sleep duration [153,157]. Mechanistically, probiotics may promote SCFA production, modulate neurotransmitter-related pathways involving GABA and 5-HT, and improve intestinal barrier integrity and inflammatory status, thereby influencing CNS function as well as sleep and emotional states [175,176,177,178].

However, the effects of these interventions are significantly influenced by strain specificity, dosage, treatment duration, and host characteristics. A randomized double-blind trial in neonates receiving antibiotic treatment found that probiotic supplementation was associated with longer sleep duration and less crying, although the differences did not reach statistical significance [179]. Therefore, large-scale and rigorously controlled clinical trials are still needed to establish standardized therapeutic protocols.

### 5.3. Lifestyle Interventions

Regular physical activity is generally associated with improved sleep quality and reduced insomnia symptoms. Exercise may promote sleep through multiple mechanisms, including thermoregulation, stress reduction, circadian reinforcement, and mood improvement [180]. Emerging evidence also suggests that exercise can reshape gut microbial composition. Regular moderate-intensity physical activity has been associated with increased microbial diversity, enrichment of SCFA-producing bacteria, and improved intestinal barrier function [181].

In addition, a recent review systematically discussed the intrinsic relationships among sleep, GM, and mind–body medicine, suggesting that mind–body interventions such as yoga, meditation, and massage may influence microbial ecology by modulating the HPA axis, ANS activity, and neuroimmune pathways, thereby improving sleep quality [182].

### 5.4. FMT

Based on preclinical evidence supporting a causal role of the GM in sleep regulation, clinical studies have begun to explore the therapeutic potential of FMT for sleep disorders. Available findings indicate that transplantation of washed microbiota from healthy donors into patients with sleep disorders may significantly shorten sleep latency, prolong sleep duration, and improve overall sleep quality while also being accompanied by changes in gut microbial structure [161]. Prospective studies have further suggested that FMT may be beneficial for post-acute COVID-19 insomnia symptoms, highlighting its therapeutic potential in complex sleep disorders [183]. Real-world studies have also shown that FMT may improve sleep and emotional outcomes in patients with chronic insomnia, together with remodeling of the GM [163]. Interestingly, in a non-insomnia subgroup from the same study, FMT was also associated with improved subjective sleep quality, suggesting that microbiota-targeted interventions may have broader sleep-regulatory effects [163].

Although these preliminary findings are encouraging, FMT remains at an exploratory stage in the field of insomnia, and rigorous randomized controlled trials are still required to confirm its efficacy and determine its long-term safety.

### 5.5. Natural Medicines

Research on the use of traditional Chinese medicine and its active constituents to improve insomnia through modulation of the MGBA is increasing. Relevant reviews have suggested that *Ziziphus jujuba* seeds, *Ganoderma lucidum*, *Poria cocos* polysaccharides, ginsenosides, and *Astragalus* polysaccharides may participate in the regulation of sleep rhythm and sleep quality by modulating gut microbial composition, promoting the production of beneficial metabolites, enhancing mucosal barrier integrity, and regulating inflammatory and neurotransmitter-related pathways [184].

In addition, one study evaluating *Lactobacillus plantarum* P8 combined with traditional Chinese herbal components (*Ziziphus jujuba*, lily bulb, *Gardenia jasminoides*, and *Poria cocos*) showed that the combined intervention significantly prolonged sleep duration, shortened sleep latency, and increased the release of 5-HT and GABA in brain tissue. Its sleep-promoting effects may be related to GM remodeling and the regulation of metabolism- and neural signaling-related pathways mediated by the microbiota [185]. These findings suggest that natural medicines, either alone or in combination with microbiota-based preparations, may represent a novel MGBA-targeted strategy for insomnia intervention.

## 6. Conclusions and Future Perspectives

**Figure 2:**
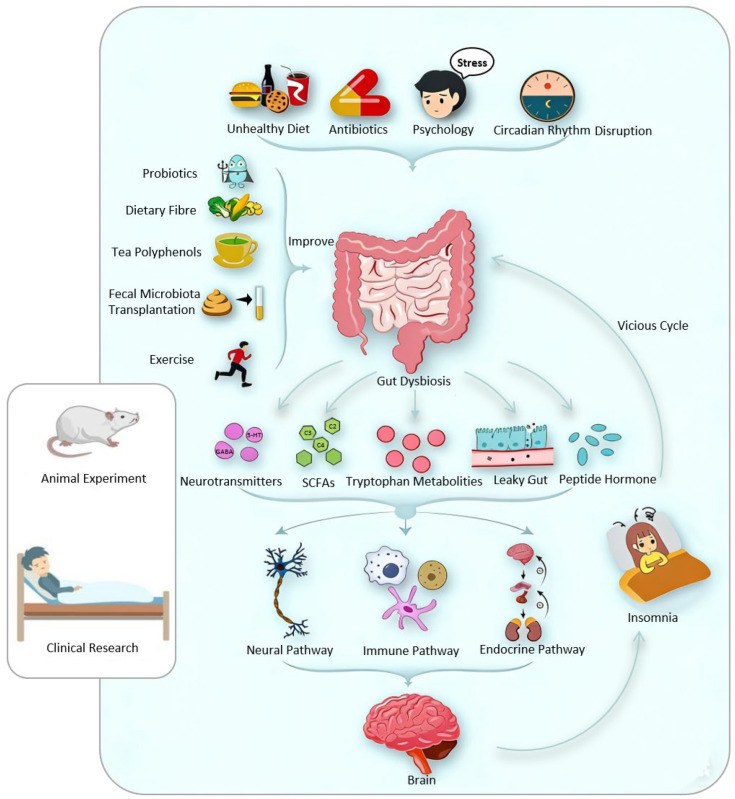
Mechanisms and microbiota-targeted interventions in insomnia. Evidence from animal experiments and clinical studies suggests that factors such as unhealthy dietary patterns, antibiotic exposure, psychological stress, and circadian rhythm disruption may induce gut microbiota (GM) dysbiosis. This dysbiosis may alter the production of microbiota-derived metabolites, including neurotransmitters, SCFAs, tryptophan metabolites, and peptide hormones, while also increasing intestinal permeability (“leaky gut”). These changes may affect the CNS through neural, immune, and endocrine pathways, thereby contributing to the development and progression of insomnia. In turn, insomnia may further disturb the GM, forming a vicious cycle. Microbiota-targeted interventions, such as high-fiber diets, tea polyphenols, regular physical activity, probiotic supplementation and fecal microbiota transplantation, may offer therapeutic potential for alleviating insomnia symptoms.

Accumulating evidence supports the MGBA as a crucial regulatory system in the development and maintenance of insomnia. Beyond traditional CNS-centric models, the MGBA integrates neural, endocrine, immune–inflammatory, and circadian pathways into a coordinated network that links peripheral microbial signals with central sleep–wake regulation. Through its influence on neurotransmitter metabolism, HPA axis activity, immune homeostasis, and circadian rhythmicity, the MGBA provides a systemic framework for understanding the complex pathophysiology of insomnia. Importantly, these pathways appear to interact in a dynamic and synergistic manner, rather than operating independently, thereby sustaining a state of hyperarousal and promoting the onset and chronicity of insomnia (Figure 2).

From a translational and clinical perspective, targeting the MGBA offers a promising and potentially modifiable approach for insomnia management. A range of microbiota-oriented interventions, including dietary modulation, prebiotics and probiotics, lifestyle interventions, FMT, and natural medicines, have demonstrated preliminary benefits in improving sleep quality and related neuropsychological outcomes (Figure 2). Notably, these strategies may act through convergent mechanisms, such as enhancing SCFA production, regulating neuroactive metabolites, restoring intestinal barrier integrity, and attenuating systemic inflammation.

Despite these advances, several important limitations should be acknowledged. Most available human studies remain cross-sectional or case–control in design, limiting causal inference. Longitudinal data capturing dynamic microbiota changes across different stages of insomnia are still scarce. In addition, interventional studies are often constrained by small sample sizes, short follow-up periods, and non-standardized outcome measures, contributing to inconsistencies across findings. Furthermore, substantial inter-individual variability related to age, sex, metabolic status, psychological factors, and baseline microbiota composition further complicates the interpretation of results. These limitations highlight existing gaps in the current evidence base and challenges in translating MGBA research into clinical practice.

Future research should prioritize large-scale, multicenter longitudinal studies and well-designed randomized controlled trials to establish causal relationships and identify clinically relevant microbial signatures. The integration of multi-omics approaches—including metagenomics, metabolomics, transcriptomics, and neuroimaging—will be essential to elucidate the dynamic interactions between the GM and central sleep-regulatory systems. Furthermore, exploring microbiota-based stratification and responder phenotypes may facilitate the development of precision medicine strategies in insomnia.

In conclusion, the MGBA not only expands the current understanding of insomnia beyond traditional neurobiological paradigms but also provides a multidimensional platform for developing innovative and individualized intervention strategies. Bridging mechanistic insights with clinical application will be critical for translating MGBA-targeted approaches into effective therapies and for establishing their role in future precision sleep medicine.

## Figures and Tables

**Table 1:** Alterations in GM Composition and Diversity in Patients with Insomnia Across Clinical Studies.

Author(s)	Year	Study Population	Diversity Changes		GM Alterations		Potential Mechanisms
Liu et al. [102]	2026	CID (Mild, Severe) vs. HC	α ↓ (S-CID vs. HC) β altered (S-CID vs. HC/ M-CID)		↓ (*Clostridium*, Ruminococcaceae); ↑ (*Bacteroides*, *Phascolarctobacterium*)		SCFA metabolism; amino acid–neurotransmitter pathways; REM sleep regulation (predicted)
Miyata et al. [103]	2025	CID	α NS; β altered		↓ *Parabacteroides* (FDR-significant)		Treatment-related microbiota shifts are associated with sleep efficiency and sleep continuity metrics.
Nie et al. [104]	2024	PI vs. HC	α NR; β altered		↓ (Firmicutes; Actinobacteria); ↑ Bacteroidetes		Metabolic/endocrine pathways (predicted).
Barone et al. [105]	2024	CID (Objective, Paradoxical) vs. HC	α NS; β altered		Objective Insomnia	↑ (Coriobacteriaceae, Erysipelotrichaceae, *Clostridium*, *Pediococcus*)	Distinct microbiota signatures discriminate insomnia subtypes, supporting a potential MGBA contribution to phenotype stratification.
					Paradoxical Insomnia	↑ (*Bacteroides*, *Staphylococcus*, *Pseudomonas*, *Proteus*)	
Zhou et al. [106]	2022	ID vs. HC	α ↓; β NS		↓ (Bacteroidaceae, Ruminococcaceae); ↑ Prevotellaceae		GM alterations correlate with serum metabolomic profiles.
Wang et al. [107]	2022	ID vs. HC	α ↓; β altered		↑ (*Lactobacillus*, *Streptococcus*, *Lactobacillus crispatus*)		Immunometabolic pathways (IL-1β ↑, TNF-α ↓)
Masyutina et al. [108]	2021	CID vs. HC	α ↓		↓ (*Faecalibacterium*, *Prevotella* 9, *Lachnospira*); ↑(*Blautia*, *Eubacterium hallii*)		Microbiota alterations correlate with inflammation (IL-6), cortisol, and sleep quality (PSQI).
Li et al. [109]	2020	ID (Acute, Chronic) vs. HC	CID	α ↓; F/B ↑; β altered	↓ (*Faecalibacterium*, *Prevotella*, *Lachnospira*); ↑ (*Blautia*, *Eubacterium hallii*)		Insomnia-related dysbiosis (reduced SCFA producers and increased pathobionts) is associated with inflammatory cytokines (notably IL-1β).
			AID	F/B ↓; β altered	↓ *Lachnospira*; ↑ *Bacteroides*		

Note. Arrows indicate the direction of change: ↑ for increase, ↓ for decrease. Abbreviations. α, alpha diversity; β, beta diversity; AID, acute insomnia disorder; CID, chronic insomnia disorder; F/B, Firmicutes/Bacteroidetes ratio; FDR, false discovery rate; GM, gut microbiota; HC, healthy controls; ID, insomnia disorder; IL, interleukin; M-CID, mild chronic insomnia disorder; MGBA, microbiota–gut–brain axis; NS, not significant; NR, not reported; PI, primary insomnia; PSQI, Pittsburgh Sleep Quality Index; REM, rapid eye movement; S-CID, severe chronic insomnia disorder; SCFA, short-chain fatty acid; TNF-α, tumor necrosis factor-alpha.

**Table 2:** Interventions for insomnia based on MGBA.

Category	Intervention	Author	Year	Study Type	Main Findings	Potential Mechanisms
Dietary interventions	Serotonin-related nutrients	Sutanto et al. [151]	2024	Clinical study	↑ sleep duration and efficiency; ↓ sleep latency; ↓ Bacteroidota; ↑ Firmicutes	Serotonin synthesis; alterations in GM
	Date seed powder supplementation	Momeniyan et al. [152]	2025	Clinical study	↑ sleep quality; ↓ anxiety- and depression-like behaviors/stress; ↓ endotoxin, cortisol, KYN, KYN/TRP ratio; ↑ IL-10, TRP, IL-10/IL-18 ratio	Regulation of tryptophan–kynurenine metabolism; anti-inflammatory effects; attenuation of metabolic endotoxemia; HPA-axis activity
Postbiotics and Probiotics	Yeast mannan	Tanihiro et al. [153]	2023	Clinical study	↑ defecation frequency and stool volumes; ↑ sleep parameters (↑ TIB, ↓ N3 latency, ↑ N3 duration); ↑ *Bacteroides thetaiotaomicron*	Alterations in GM; potential involvement of microbial metabolites (e.g., propionate, GABA)
	GOS/PDX prebiotic diet	Thompson et al. [154]	2021	Animal study	↑ sleep–wake rhythm realignment; ↑ *Parabacteroides distasonis*, *Ruminiclostridium 5*, *Clostridium leptum*; ↓ fecal secondary bile acids; ↑α	Modulation of GM; bile acid-related pathways; associated with circadian rhythm regulation
	BLa80 supplementation	Liu et al. [155]	2025	Clinical study	↓ PSQI; ↓ Proteobacteria; ↑ Bacteroidetes, *Fusicatenibacter*, *Parabacteroides*	Modulation of GM; predicted changes in microbial metabolic pathways (purine metabolism; glycolysis/gluconeogenesis; arginine biosynthesis pathways)
	NVP-1704	Lee et al. [156]	2021	Clinical study	↓ SRI, BAI, BDI-II, PSQI, ISI; ↓ IL-6; ↑ Bifidobacteriaceae, Lactobacillaceae; ↓ Enterobacteriaceae; ↑ α; β altered	Modulation of GM; inflammatory markers
	*Lacticaseibacillus paracasei* 207-27	Li et al. [157]	2024	Clinical study	↓ PSQI; ↓ saliva cortisol; ↑ Bacteroidota, *Bacteroides*, *Megamonas*; ↓ F/B ratio, *Escherichia-Shigella*; ↑ SCFAs	Modulation of GM and metabolites; potential involvement of neuroendocrine-related changes
Lifestyle interventions	Physical activity	Magzal et al. [158]	2022	Clinical study	↑ Sleep efficiency; ↑ Erysipelotrichaceae, Peptococcaceae, *Peptococcus*, *Catenibacterium*	Associations between physical activity, GM composition, and sleep parameters
	Long-Term Exercise	Zheng et al. [159]	2025	Animal study	Stable GM structure (↑ *Lachnospiraceae_NK4A136_group*, *Lachnospiraceae-UCG-006*, β altered); ↑ butyrate; ↓ LPS, IL-6, TLR4, NF-κB	Modulation of GM and metabolites; association with inflammatory markers
Natural Medicines	Ziziphi Spinosae Semen	Bian et al. [160]	2025	Animal study	↑ sleep status, cognitive ability; ↓ neuronal damage; ↑ GABA; ↓ Glu; ↑ GAD67, GABRA1, GABRG2; ↓ GluR1, NMDAR1, mGluR5; ↑ *Lactobacillus johnsonii*	Modulation of GM; GABA/Glu balance regulation; GABAergic signaling pathway; glutamatergic signaling pathway
FMT	Washed microbiota transplantation	He et al. [161]	2024	Clinical study	↑ sleep quality; ↓ sleep latency; ↑ general health, vitality, social function and mental health; ↓ PSQI	Modulation of GM; involvement of the MGBA
	*Bifidobacterium longum* P77 and *Lactiplantibacillus plantarum* P72	Baek et al. [162]	2025	Animal study	↓ depression-, anxiety-, and sleeplessness-like behaviors; ↓ CORT, TNF-α, NF-κB; ↑ IL-10, GABA, 5-HT	Modulation of GM; GABAergic and serotonergic systems; association with inflammatory pathway
	Fecal microbiota transplantation	Fang et al. [163]	2023	Clinical study	↓ ISI, PSQI, SAS, SDS; ↑ life quality, sleep quality; ↑ *Lactobacillus*, *Bifidobacterium*, *Turicibacter*, *Anaerostipes*, *Eisenbergiella*	Modulation of GM and associated improvements in sleep and psychological parameters

Note. The mechanisms listed represent potential pathways proposed in the original studies and may not reflect experimentally confirmed causal relationships. Arrows indicate the direction of change: ↑ for increase, ↓ for decrease. Abbreviations. 5-HT, 5-hydroxytryptamine; BAI, Beck Anxiety Inventory; BDI-II, Beck Depression Inventory-II; CORT, corticosterone; FMT, fecal microbiota transplantation; GABA, gamma-aminobutyric acid; GABRA1, gamma-aminobutyric acid type A receptor subunit alpha1; GABRG2, gamma-aminobutyric acid type A receptor subunit gamma2; GAD67, glutamate decarboxylase 67; Glu, glutamate; GluR1, glutamate receptor 1; GOS, galactooligosaccharide; HPA axis, hypothalamic–pituitary–adrenal axis; ISI, Insomnia Severity Index; KYN, kynurenine; LPS, lipopolysaccharide; mGluR5, metabotropic glutamate receptor 5; N3, non-rapid eye movement stage 3 sleep; NF-κB, nuclear factor kappa-B; NMDAR1, N-methyl-D-aspartate receptor 1; PDX, polydextrose; SAS, Self-Rating Anxiety Scale; SDS, Self-Rating Depression Scale; SRI, Sleep Regulation Index; TLR4, Toll-like receptor 4; TRP, tryptophan; TIB, time in bed.

## Data Availability

The data that support the findings of this study are available from the corresponding author, C.B., upon reasonable request.

## References

[1] Johnson D.A., Stranges S. (2025). Time to act: Dismantling social barriers to healthy sleep across the life-course. Eur. J. Public Health.

[2] Benjafield A.V., Sert Kuniyoshi F.H., Malhotra A., Martin J.L., Morin C.M., Maurer L.F., Cistulli P.A., Pépin J.L., Wickwire E.M. (2025). Estimation of the global prevalence and burden of insomnia: A systematic literature review-based analysis. Sleep Med. Rev..

[3] Endomba F.T., Tchebegna P.Y., Chiabi E., Angong Wouna D.L., Guillet C., Chauvet-Gélinier J.C. (2023). Epidemiology of insomnia disorder in older persons according to the Diagnostic and Statistical Manual of Mental Disorders: A systematic review and meta-analysis. Eur. Geriatr. Med..

[4] Yoo J., Slavish D., Dietch J.R., Kelly K., Ruggero C., Taylor D.J. (2023). Daily reactivity to stress and sleep disturbances: Unique risk factors for insomnia. Sleep.

[5] van Straten A., Weinreich K.J., Fábián B., Reesen J., Grigori S., Luik A.I., Harrer M., Lancee J. (2025). The prevalence of insomnia disorder in the general population: A meta-analysis. J. Sleep Res..

[6] Porcheret K., Hopstock L.A., Nilsen K.B. (2024). Prevalence of insomnia in a general adult population cohort using different diagnostic criteria: The seventh survey of the Tromsø study 2015-2016. Sleep Med..

[7] Dressle R.J., Riemann D. (2023). Hyperarousal in insomnia disorder: Current evidence and potential mechanisms. J. Sleep Res..

[8] Zhu W., Huang L., Cheng H., Li N., Zhang B., Dai W., Wu X., Zhang D., Feng W., Li S. (2024). GABA and its receptors’ mechanisms in the treatment of insomnia. Heliyon.

[9] Akkaoui M.A., Palagini L., Geoffroy P.A. (2023). Sleep immune cross talk and insomnia. Adv. Exp. Med. Biol..

[10] Hartstein L.E., Grandner M.A., Diniz Behn C. (2025). Sleep irregularity and circadian rhythmicity: Implications for health and well-being. Curr. Sleep Med. Rep..

[11] Tang N.K.Y., Saconi B., Jansson-Fröjmark M., Ong J.C., Carney C.E. (2023). Cognitive factors and processes in models of insomnia: A systematic review. J. Sleep Res..

[12] Freeman D., Sheaves B., Waite F., Harvey A.G., Harrison P.J. (2020). Sleep disturbance and psychiatric disorders. Lancet Psychiatry.

[13] Fornaro M., Caiazza C., De Simone G., Rossano F., De Bartolomeis A. (2024). Insomnia and related mental health conditions: Essential neurobiological underpinnings towards reduced polypharmacy utilization rates. Sleep Med..

[14] Zhang Y., Jiang X., Liu J., Lang Y., Liu Y. (2021). The association between insomnia and the risk of metabolic syndrome: A systematic review and meta-analysis. J. Clin. Neurosci..

[15] Zhang X., Sun Y., Ye S., Huang Q., Zheng R., Li Z., Yu F., Zhao C., Zhang M., Zhao G. (2024). Associations between insomnia and cardiovascular diseases: A meta-review and meta-analysis of observational and Mendelian randomization studies. J. Clin. Sleep Med..

[16] Lu S., Zhao Q., Guan Y., Sun Z., Li W., Guo S., Zhang A. (2024). The communication mechanism of the gut-brain axis and its effect on central nervous system diseases: A systematic review. Biomed. Pharmacother..

[17] Lin Z., Jiang T., Chen M., Ji X., Wang Y. (2024). Gut microbiota and sleep: Interaction mechanisms and therapeutic prospects. Open Life Sci..

[18] Wang Y., Xie S., Chen S., Li C., Chan Y.L., Chan N.Y., Wing Y.K., Chan F.K.L., Su Q., Ng S.C. (2025). The role of gut microbiota in insomnia: A systematic review of case-control studies. Life.

[19] Vemuri R., Shankar E.M., Chieppa M., Eri R., Kavanagh K. (2020). Beyond just bacteria: Functional biomes in the gut ecosystem including virome, mycobiome, archaeome and helminths. Microorganisms.

[20] Dmytriv T.R., Storey K.B., Lushchak V.I. (2024). Intestinal barrier permeability: The influence of gut microbiota, nutrition, and exercise. Front. Physiol..

[21] Kim S., Ndwandwe C., Devotta H., Kareem L., Yao L., O’Mahony L. (2025). Role of the microbiome in regulation of the immune system. Allergol. Int..

[22] Michaudel C., Sokol H. (2020). The gut microbiota at the service of immunometabolism. Cell Metab..

[23] Safarchi A., Al-Qadami G., Tran C.D., Conlon M. (2025). Understanding dysbiosis and resilience in the human gut microbiome: Biomarkers, interventions, and challenges. Front. Microbiol..

[24] Hu X., Yu C., He Y., Zhu S., Wang S., Xu Z., You S., Jiao Y., Liu S.L., Bao H. (2024). Integrative metagenomic analysis reveals distinct gut microbial signatures related to obesity. BMC Microbiol..

[25] Piccioni A., Rosa F., Mannucci S., Manca F., Merra G., Chiloiro S., Candelli M., Covino M., Gasbarrini A., Franceschi F. (2023). Gut microbiota, LADA, and type 1 diabetes mellitus: An evolving relationship. Biomedicines.

[26] Wang Y., Wei J., Zhang W., Doherty M., Zhang Y., Xie H., Li W., Wang N., Lei G., Zeng C. (2022). Gut dysbiosis in rheumatic diseases: A systematic review and meta-analysis of 92 observational studies. EBioMedicine.

[27] Cao Y., Cheng Y., Pan W., Diao J., Sun L., Meng M. (2025). Gut microbiota variations in depression and anxiety: A systematic review. BMC Psychiatry.

[28] Morais L.H., Schreiber H.L., Mazmanian S.K. (2021). The gut microbiota–brain axis in behaviour and brain disorders. Nat. Rev. Microbiol..

[29] Duan H., Cai X., Luan Y., Yang S., Yang J., Dong H., Zeng H., Shao L. (2021). Regulation of the autonomic nervous system on intestine. Front. Physiol..

[30] Cao Y., Li R., Bai L. (2024). Vagal sensory pathway for the gut-brain communication. Semin. Cell Dev. Biol..

[31] Barton J.R., Londregan A.K., Alexander T.D., Entezari A.A., Covarrubias M., Waldman S.A. (2023). Enteroendocrine cell regulation of the gut-brain axis. Front. Neurosci..

[32] Onimus O., Arrivet F., Le Borgne T., Perez S., Castel J., Ansoult A., Bertrand B., Mashhour N., de Almeida C., Bui L.C. (2026). The gut-brain vagal axis governs mesolimbic dopamine dynamics and reward events. Sci. Adv..

[33] Onimus O., Arrivet F., Souza I.N.O., Bertrand B., Castel J., Luquet S., Mothet J.P., Heck N., Gangarossa G. (2024). The gut-brain vagal axis scales hippocampal memory processes and plasticity. Neurobiol. Dis..

[34] Cook T.M., Gavini C.K., Jesse J., Aubert G., Gornick E., Bonomo R., Gautron L., Layden B.T., Mansuy-Aubert V. (2021). Vagal neuron expression of the microbiota-derived metabolite receptor, free fatty acid receptor (FFAR3), is necessary for normal feeding behavior. Mol. Metab..

[35] Wu X., Li J.Y., Lee A., Lu Y.X., Zhou S.Y., Owyang C. (2020). Satiety induced by bile acids is mediated via vagal afferent pathways. JCI Insight.

[36] Hwang Y.K., Oh J.S. (2025). Interaction of the vagus nerve and serotonin in the gut-brain axis. Int. J. Mol. Sci..

[37] Niesler B., Kuerten S., Demir I.E., Schäfer K.H. (2021). Disorders of the enteric nervous system—A holistic view. Nat. Rev. Gastroenterol. Hepatol..

[38] Linden D.R., Sharkey K.A. (2025). The enteric nervous system. Curr. Biol..

[39] Sharkey K.A., Mawe G.M. (2023). The enteric nervous system. Physiol. Rev..

[40] Geng Z.H., Zhu Y., Li Q.L., Zhao C., Zhou P.H. (2022). Enteric nervous system: The bridge between the gut microbiota and neurological disorders. Front. Aging Neurosci..

[41] Harding E.K., Fung S.W., Bonin R.P. (2020). Insights into spinal dorsal horn circuit function and dysfunction using optical approaches. Front. Neural Circuits.

[42] Münzberg H., Berthoud H.R., Neuhuber W.L. (2023). Sensory spinal interoceptive pathways and energy balance regulation. Mol. Metab..

[43] De Preter C.C., Heinricher M.M. (2024). The ‘in’s and out’s’ of descending pain modulation from the rostral ventromedial medulla. Trends Neurosci..

[44] Nürnberger F., Ott D., Claßen R., Rummel C., Roth J., Leisengang S. (2022). Systemic lipopolysaccharide challenge induces inflammatory changes in rat dorsal root ganglia: An ex vivo study. Int. J. Mol. Sci..

[45] Grundeken E., El Aidy S. (2025). Enteroendocrine cells: The gatekeepers of microbiome-gut-brain communication. npj Biofilms Microbiomes.

[46] Xu M., Zhou E.Y., Shi H. (2025). Tryptophan and its metabolite serotonin impact metabolic and mental disorders via the brain-gut-microbiome axis: A focus on sex differences. Cells.

[47] Moaket O.S., Obaid S.E., Obaid F.E., Shakeeb Y.A., Elsharief S.M., Tania A., Darwish R., Butler A.E., Moin A.S.M. (2025). GLP-1 and the degenerating brain: Exploring mechanistic insights and therapeutic potential. Int. J. Mol. Sci..

[48] Atanga R., Singh V., In J.G. (2023). Intestinal enteroendocrine cells: Present and future druggable targets. Int. J. Mol. Sci..

[49] Roy A., Dawson V.L., Dawson T.M. (2025). From metabolism to mind: The expanding role of the GLP-1 receptor in neurotherapeutics. Neurotherapeutics.

[50] Rusch J.A., Layden B.T., Dugas L.R. (2023). Signalling cognition: The gut microbiota and hypothalamic-pituitary-adrenal axis. Front. Endocrinol..

[51] Bertollo A.G., Santos C.F., Bagatini M.D., Ignácio Z.M. (2025). Hypothalamus-pituitary-adrenal and gut-brain axes in biological interaction pathway of the depression. Front. Neurosci..

[52] Madison A.A., Bailey M.T. (2024). Stressed to the core: Inflammation and intestinal permeability link stress-related gut microbiota shifts to mental health outcomes. Biol. Psychiatry.

[53] Shoji H., Maeda Y., Miyakawa T. (2024). Chronic corticosterone exposure causes anxiety- and depression-related behaviors with altered gut microbial and brain metabolomic profiles in adult male C57BL/6J mice. Mol. Brain.

[54] Rivera C.A., Lennon-Duménil A.M. (2023). Gut immune cells and intestinal niche imprinting. Semin. Cell Dev. Biol..

[55] Bostick J.W., Schonhoff A.M., Mazmanian S.K. (2022). Gut microbiome-mediated regulation of neuroinflammation. Curr. Opin. Immunol..

[56] Park J.C., Chang L., Kwon H.K., Im S.H. (2025). Beyond the gut: Decoding the gut-immune-brain axis in health and disease. Cell. Mol. Immunol..

[57] Peña-Durán E., García-Galindo J.J., López-Murillo L.D., Huerta-Huerta A., Balleza-Alejandri L.R., Beltrán-Ramírez A., Anaya-Ambriz E.J., Suárez-Rico D.O. (2025). Microbiota and inflammatory markers: A review of their interplay, clinical implications, and metabolic disorders. Int. J. Mol. Sci..

[58] Beltran-Velasco A.I., Clemente-Suárez V.J. (2025). Impact of peripheral inflammation on blood-brain barrier dysfunction and its role in neurodegenerative diseases. Int. J. Mol. Sci..

[59] Yang G., Xu X., Gao W., Wang X., Zhao Y., Xu Y. (2025). Microglia-orchestrated neuroinflammation and synaptic remodeling: Roles of pro-inflammatory cytokines and receptors in neurodegeneration. Front. Cell. Neurosci..

[60] Keszthelyi D. (2024). Cytokine-responsive vagal afferents and the nucleus of the solitary tract: Orchestrators of immune function. Gastroenterology.

[61] Kelly M.J., Breathnach C., Tracey K.J., Donnelly S.C. (2022). Manipulation of the inflammatory reflex as a therapeutic strategy. Cell Rep. Med..

[62] Morys J., Małecki A., Nowacka-Chmielewska M. (2024). Stress and the gut-brain axis: An inflammatory perspective. Front. Mol. Neurosci..

[63] Xu J., Wang B., Ao H. (2025). Corticosterone effects induced by stress and immunity and inflammation: Mechanisms of communication. Front. Endocrinol..

[64] Van Someren E.J.W. (2021). Brain mechanisms of insomnia: New perspectives on causes and consequences. Physiol. Rev..

[65] Palagini L., Geoffroy P.A., Miniati M., Perugi G., Biggio G., Marazziti D., Riemann D. (2022). Insomnia, sleep loss, and circadian sleep disturbances in mood disorders: A pathway toward neurodegeneration and neuroprogression? A theoretical review. CNS Spectr..

[66] Guo J., Guo J., Rao X., Zhang R., Li Q., Zhang K., Ma S., Zhao J., Ji C. (2024). Exploring the pathogenesis of insomnia and acupuncture intervention strategies based on the microbiota-gut-brain axis. Front. Microbiol..

[67] Gao K., Mu C.L., Farzi A., Zhu W.Y. (2020). Tryptophan metabolism: A link between the gut microbiota and brain. Adv. Nutr..

[68] Rentschler K.M., Milosavljevic S., Baratta A.M., Wright C.J., Piroli M.V., Tentor Z., Valafar H., O’Reilly C., Pocivavsek A. (2024). Reducing brain kynurenic acid synthesis precludes kynurenine-induced sleep disturbances. J. Sleep Res..

[69] Han J., Zhang L., Lyu Y., Zhai Y., Wu W., Qin Y., Ma L., Zhuo L., Guo Y., Wang X. (2026). Indole-3-acetic acid exerts protective effects on sleep deprivation-induced cognitive impairment. Neuropharmacology.

[70] Murray M., Barlow C.K., Blundell S., Buecking M., Gibbon A., Goeckener B., Kaminskas L.M., Leitner P., Selby-Pham S., Sinclair A. (2023). Demonstrating a link between diet, gut microbiota and brain: 14C radioactivity identified in the brain following gut microbial fermentation of 14C-radiolabeled tyrosine in a pig model. Front. Nutr..

[71] Hepsomali P., Groeger J.A., Nishihira J., Scholey A. (2020). Effects of oral gamma-aminobutyric acid (GABA) administration on stress and sleep in humans: A systematic review. Front. Neurosci..

[72] Li S., Li Y., Xue C., Zhang Y., Tong T., Ouyang Z., Liu D., Cai J., Sun H. (2025). Progress in research on the mechanism of GABA in improving sleep. Foods.

[73] Hamamah S., Aghazarian A., Nazaryan A., Hajnal A., Covasa M. (2022). Role of microbiota-gut-brain axis in regulating dopaminergic signaling. Biomedicines.

[74] Dressle R.J., Feige B., Spiegelhalder K., Schmucker C., Benz F., Mey N.C., Riemann D. (2022). HPA axis activity in patients with chronic insomnia: A systematic review and meta-analysis of case-control studies. Sleep Med. Rev..

[75] Kalmbach D.A., Fernandez-Mendoza J., Drake C.L. (2023). Stress and sleep reactivity increase risk for insomnia: Highlighting the dynamic interplay between sleep-wake regulation and stress responsivity. Sleep.

[76] Tofani G.S.S., Leigh S.J., Gheorghe C.E., Bastiaanssen T.F.S., Wilmes L., Sen P., Clarke G., Cryan J.F. (2025). Gut microbiota regulates stress responsivity via the circadian system. Cell Metab..

[77] Tan H.E. (2023). The microbiota-gut-brain axis in stress and depression. Front. Neurosci..

[78] Qiao Y., Cheng R., Li X., Zheng H., Guo J., Wei L., Gao T., Bi H. (2025). Plateau environment, gut microbiota, and depression: A possible concealed connection?. Curr. Issues Mol. Biol..

[79] Jiang C., Chen Y., Sun T. (2025). From the gut to the brain, mechanisms and clinical applications of γ-aminobutyric acid (GABA) on the treatment of anxiety and insomnia. Front. Neurosci..

[80] Liu J., Jing C., Guo Y., Shang Z., Zhang B., Zhou X., Zhang J., Lian G., Tian F., Li L. (2025). The central signaling pathways related to metabolism-regulating hormones of the gut-brain axis: A review. J. Transl. Med..

[81] Huang S., Yu S., Zhang W., Qi D., Pei X., Lu D., Ba M., Xuan S., Huang D., Yang J. (2026). Sleep deprivation disrupts lacrimal gland homeostasis via hypothalamic-pituitary-adrenal axis and gut dysbiosis in mice. Commun. Biol..

[82] Feuth T. (2024). Interactions between sleep, inflammation, immunity and infections: A narrative review. Immun. Inflamm. Dis..

[83] Ballesio A. (2023). Where does inflammation in insomnia come from? and does it matter for comorbidity?. Sleep.

[84] Kinlein S.A., Karatsoreos I.N. (2020). The hypothalamic-pituitary-adrenal axis as a substrate for stress resilience: Interactions with the circadian clock. Front. Neuroendocrinol..

[85] Zhang N., Park K., Chung S., Yim Y.S. (2025). IL-1b and TNF-a-driven sleep alterations: Neuroimmune mechanisms and behavioral implications. Brain Behav. Immun. Health.

[86] Das A., Mohan Raj P.S., Vishwakarma L.C., Jha P.K., Bouaouda H. (2024). Sleep and neuroinflammation. Circadian Rhythms, Sleep and Inflammation.

[87] Du Y., He C., An Y., Huang Y., Zhang H., Fu W., Wang M., Shan Z., Xie J., Yang Y. (2024). The role of short chain fatty acids in inflammation and body health. Int. J. Mol. Sci..

[88] Grondin J.A., Khan W.I. (2023). Emerging roles of gut serotonin in regulation of immune response, microbiota composition and intestinal inflammation. J. Can. Assoc. Gastroenterol..

[89] Wang Y., Zhang Y., Wang W., Zhang Y., Dong X., Liu Y. (2025). Diverse physiological roles of kynurenine pathway metabolites: Updated implications for health and disease. Metabolites.

[90] Addae J.I., Stone T.W., Kostrzewa R.M. (2022). Quinolinic acid and related excitotoxins: Mechanisms of neurotoxicity and disease relevance. Handbook of Neurotoxicity.

[91] Bautista J., Ojeda-Mosquera S., Altamirano-Colina A., Hidalgo-Tinoco C., Di Capua Delgado M., López-Cortés A. (2025). Bidirectional interactions between circadian rhythms and the gut microbiome. Appl. Microbiol. Biotechnol..

[92] Heddes M., Altaha B., Niu Y., Reitmeier S., Kleigrewe K., Haller D., Kiessling S. (2022). The intestinal clock drives the microbiome to maintain gastrointestinal homeostasis. Nat. Commun..

[93] Firoozi D., Masoumi S.J., Hosseini Asl S.M.K., Nekooeian A.A., Zare M., Tanideh N. (2024). Effects of short-chain fatty acid-butyrate supplementation on expression of circadian-clock genes, sleep quality, and inflammation in patients with active ulcerative colitis: A double-blind randomized controlled trial. Lipids Health Dis..

[94] Fawad J.A., Luzader D.H., Hanson G.F., Moutinho T.J., McKinney C.A., Mitchell P.G., Brown-Steinke K., Kumar A., Park M., Lee S. (2022). Histone deacetylase inhibition by gut microbe-generated short-chain fatty acids entrains intestinal epithelial circadian rhythms. Gastroenterology.

[95] Bello A.T., Sarafian M.H., Wimborne E.A., Middleton B., Revell V.L., Raynaud F.I., Chowdhury N.R., van der Veen D.R., Skene D.J., Swann J.R. (2024). Exposing 24-hour cycles in bile acids of male humans. Nat. Commun..

[96] Yuan R.K., Zitting K.M. (2025). Sleep and circadian effects on the incretin system. Curr. Sleep Med. Rep..

[97] Ahmadi S., Taghizadieh M., Mehdizadehfar E., Hasani A., Khalili Fard J., Feizi H., Hamishehkar H., Ansarin M., Yekani M., Memar M.Y. (2024). Gut microbiota in neurological diseases: Melatonin plays an important regulatory role. Biomed. Pharmacother..

[98] Iesanu M.I., Zahiu C.D.M., Dogaru I.A., Chitimus D.M., Pircalabioru G.G., Voiculescu S.E., Isac S., Galos F., Pavel B., O’Mahony S.M. (2022). Melatonin–microbiome two-sided interaction in dysbiosis-associated conditions. Antioxidants.

[99] Ogawa Y., Miyoshi C., Obana N., Yajima K., Hotta-Hirashima N., Ikkyu A., Kanno S., Soga T., Fukuda S., Yanagisawa M. (2020). Gut microbiota depletion by chronic antibiotic treatment alters the sleep/wake architecture and sleep EEG power spectra in mice. Sci. Rep..

[100] Wang Z., Wang Z., Lu T., Yuan G., Chen W., Jin J., Jiang X., Yan W., Yuan K., Zou G. (2025). Gut microbiota regulate insomnia-like behaviors via gut-brain metabolic axis. Mol. Psychiatry.

[101] Cai S., Li Z., Bai J., Ding Y., Liu R., Fang L., Hou D., Zhang S., Wang X., Wang Y. (2025). Optimized oxygen therapy improves sleep deprivation-induced cardiac dysfunction through gut microbiota. Front. Cell. Infect. Microbiol..

[102] Liu Y., Cai Y., Shi X., Fan M., Zhang X., Lin J., Fan X., Liu B., Pan J. (2026). Distinct gut microbiota profiles reflect severity in chronic insomnia disorder. Brain Behav..

[103] Miyata S., Iwamoto K., Ito M., Okada I., Matsuyama N., Fujimoto A., Kogo Y., Nishiwaki H., Ueyama J., Ohno K. (2025). Gut microbiome composition changes during insomnia treatment with Lemborexant. Nat. Sci. Sleep.

[104] Nie L., Xiang Q., Lin Y., Xu Y., Wen W., Deng Y., Chen J., Zhu X., Xie L., Wu Z. (2024). Correlation between symptoms and cognitive function changes in patients with primary insomnia and pathways in gut microbiota. Biochem. Biophys. Rep..

[105] Barone M., Martucci M., Sciara G., Conte M., Medina L.S.J., Iattoni L., Miele F., Fonti C., Franceschi C., Brigidi P. (2024). Towards a personalized prediction, prevention and therapy of insomnia: Gut microbiota profile can discriminate between paradoxical and objective insomnia in post-menopausal women. EPMA J..

[106] Zhou J., Wu X., Li Z., Zou Z., Dou S., Li G., Yan F., Chen B., Li Y. (2022). Alterations in gut microbiota are correlated with serum metabolites in patients with insomnia disorder. Front. Cell. Infect. Microbiol..

[107] Wang Q., Chen B., Sheng D., Yang J., Fu S., Wang J., Zhao C., Wang Y., Gai X., Wang J. (2022). Multiomics analysis reveals aberrant metabolism and immunity linked gut microbiota with insomnia. Microbiol. Spectr..

[108] Masyutina A.A., Gumenyuk L.N., Fatovenko Y.V., Sorokina L.E., Bayramova S.S., Alekseenko A.I., Shavrov Y.V., Romanova A.A., Seydametova D.I. (2021). Changes in gut microbiota composition and their associations with cortisol, melatonin and interleukin 6 in patients with chronic insomnia. Bull. Russ. State Med. Univ..

[109] Li Y., Zhang B., Zhou Y., Wang D., Liu X., Li L., Wang T., Zhang Y., Jiang M., Tang H. (2020). Gut microbiota changes and their relationship with inflammation in patients with acute and chronic insomnia. Nat. Sci. Sleep.

[110] Yerlikaya F.H., Onmaz D.E., Selvi Y., Topkafa M., Sivrikaya A., Kaya S., Akdağ F. (2025). Insomnia patients have a poor intestinal prognosis: Accompanied by microbiota-derived short chain fatty acids, diet and zonulin. J. Psychiatr. Res..

[111] Zhang C., Sheng Q., Wang Y., Shen Q., Zhai Y., Hu D., Zhang N., Wang Z., Yin X., Li D. (2025). Characteristics and influencing factors of gut microbiota in population with sleep disorders. Front. Microbiol..

[112] Lin W., Yang Y., Zhu Y., Pan R., Liu C., Pan J. (2024). Linking gut microbiota, oral microbiota, and serum metabolites in insomnia disorder: A preliminary study. Nat. Sci. Sleep.

[113] Onu A., Tutu A., Trofin D.M., Onu I., Galaction A.I., Onita C.A., Iordan D.A., Matei D.V. (2026). Diet, physical exercise, and gut microbiota modulation in metabolic syndrome: A narrative review. Life.

[114] Yang Y., Hernandez M.C., Chitre S., Jobin C. (2025). Emerging roles of modern lifestyle factors in microbiome stability and functionality. Curr. Clin. Micro. Rpt..

[115] Garg K., Mohajeri M.H. (2024). Potential effects of the most prescribed drugs on the microbiota-gut-brain-axis: A review. Brain Res. Bull..

[116] Grasa-Ciria D., Couto S., Samatán E., Martínez-Jarreta B., Cenit M.D.C., Iguacel I. (2025). Disrupted rhythms, disrupted microbes: A systematic review of shift work and gut microbiota alterations. Nutrients.

[117] Kasarello K., Cudnoch-Jedrzejewska A., Czarzasta K. (2023). Communication of gut microbiota and brain via immune and neuroendocrine signaling. Front. Microbiol..

[118] Tian B., Geng Y., Wang P., Cai M., Neng J., Hu J., Xia D., Cao W., Yang K., Sun P. (2022). Ferulic acid improves intestinal barrier function through altering gut microbiota composition in high-fat diet-induced mice. Eur. J. Nutr..

[119] Arnone D., Chabot C., Heba A.C., Kökten T., Caron B., Hansmannel F., Dreumont N., Ananthakrishnan A.N., Quilliot D., Peyrin-Biroulet L. (2022). Sugars and gastrointestinal health. Clin. Gastroenterol. Hepatol..

[120] Vignesh S.D., Vijayakumar T.M., Siddhu N.S.S. (2024). Impact of food intake and sleep disturbances on gut microbiota. Cureus.

[121] St-Onge M.P., Cherta-Murillo A., Darimont C., Mantantzis K., Martin F.P., Owen L. (2023). The interrelationship between sleep, diet, and glucose metabolism. Sleep Med. Rev..

[122] Lavrinienko A., Greppi A., Häcki S., Paramonova D., Bokulich N.A. (2025). Impacts of food additive sweeteners and emulsifiers on the gut microbiome: Research trends and future directions. Trends Food Sci. Technol..

[123] Qiu L., Gong F., Wu J., You D., Zhao Y., Xu L., Cao X., Bao F. (2022). Exercise interventions improved sleep quality through regulating intestinal microbiota composition. Int. J. Environ. Res. Public Health.

[124] Xu L., Li W., Ling L., Zhang Z., Cui Z., Ge J., Wang Y., Meng Q., Wang Y., Liu K. (2023). A sedentary lifestyle changes the composition and predicted functions of the gut bacterial and fungal microbiota of subjects from the same company. Curr. Microbiol..

[125] Lu Y., Liu M., Chen S., Liu X. (2025). Associations of physical activity and sedentary behavior with insomnia in middle-aged and older adults: A cross-sectional study. Front. Public Health.

[126] McCullar K.S., Barker D.H., McGeary J.E., Saletin J.M., Gredvig-Ardito C., Swift R.M., Carskadon M.A. (2024). Altered sleep architecture following consecutive nights of presleep alcohol. Sleep.

[127] Qamar N., Castano D., Patt C., Chu T., Cottrell J., Chang S.L. (2019). Meta-analysis of alcohol induced gut dysbiosis and the resulting behavioral impact. Behav. Brain Res..

[128] Engen P.A., Green S.J., Voigt R.M., Forsyth C.B., Keshavarzian A. (2015). The gastrointestinal microbiome: Alcohol effects on the composition of intestinal microbiota. Alcohol Res. Curr. Rev..

[129] Zheng Y.B., Huang Y.T., Gong Y.M., Li M.Z., Zeng N., Wu S.L., Zhang Z.B., Tian S.S., Yuan K., Liu X.X. (2024). Association of lifestyle with sleep health in general population in China: A cross-sectional study. Transl. Psychiatry.

[130] Yu X., Qiu Z., Zeng X., Zhang S., Tang J., Wu Y., Zhang L., Huo X., Liu C., Liu D. (2025). Influences of PM2.5 on gut physiology, microbiota and metabolites. Ecotoxicol. Environ. Saf..

[131] Touitou Y., Perlemuter G., Touitou C. (2025). Shift work, gut dysbiosis, and circadian misalignment: The combined impact of nighttime light exposure, nutrients, and microbiota rhythmicity. Chronobiol. Int..

[132] James K.A., Stromin J.I., Steenkamp N., Combrinck M.I. (2023). Understanding the relationships between physiological and psychosocial stress, cortisol and cognition. Front. Endocrinol..

[133] Rábago-Monzón Á.R., Osuna-Ramos J.F., Armienta-Rojas D.A., Camberos-Barraza J., Camacho-Zamora A., Magaña-Gómez J.A., De la Herrán-Arita A.K. (2025). Stress-induced sleep dysregulation: The roles of astrocytes and microglia in neurodegenerative and psychiatric disorders. Biomedicines.

[134] Mao T., Guo B., Rao H. (2024). Unraveling the complex interplay between insomnia, anxiety, and brain networks. Sleep.

[135] Vargas I., Nguyen A.M., Haeffel G.J., Drake C.L. (2020). A negative cognitive style is associated with greater insomnia and depression symptoms: The mediating role of sleep reactivity. J. Affect. Disord. Rep..

[136] Zhang J., Xiang S., Li X., Tang Y., Hu Q. (2024). The impact of stress on sleep quality: A mediation analysis based on longitudinal data. Front. Psychol..

[137] Ge D. (2024). The relationship between loneliness and sleep quality: Multiple chain mediating roles. Curr. Psychol..

[138] Tanaka A., Sanada K., Miyaho K., Tachibana T., Kurokawa S., Ishii C., Noda Y., Nakajima S., Fukuda S., Mimura M. (2023). The relationship between sleep, gut microbiota, and metabolome in patients with depression and anxiety: A secondary analysis of the observational study. PLoS ONE.

[139] Yu D.J., Recchia F., Bernal J.D.K., Yu A.P., Fong D.Y., Li S.X., Chan R.N.Y., Hu X., Siu P.M. (2023). Effectiveness of exercise, cognitive behavioral therapy, and pharmacotherapy on improving sleep in adults with chronic insomnia: A systematic review and network meta-analysis of randomized controlled trials. Healthcare.

[140] Mirchandaney R., Barete R., Asarnow L.D. (2022). Moderators of cognitive behavioral treatment for insomnia on depression and anxiety outcomes. Curr. Psychiatry Rep..

[141] Fishbein S.R.S., Mahmud B., Dantas G. (2023). Antibiotic perturbations to the gut microbiome. Nat. Rev. Microbiol..

[142] Elvers K.T., Wilson V.J., Hammond A., Duncan L., Huntley A.L., Hay A.D., van der Werf E.T. (2020). Antibiotic-induced changes in the human gut microbiota for the most commonly prescribed antibiotics in primary care in the UK: A systematic review. BMJ Open.

[143] Guarner F., Bustos Fernandez L., Cruchet S., Damião A., Maruy Saito A., Riveros Lopez J.P., Rodrigues Silva L., Valdovinos Diaz M.A. (2024). Gut dysbiosis mediates the association between antibiotic exposure and chronic disease. Front. Med..

[144] Bibi A., Zhang F., Shen J., Din A.U., Xu Y. (2025). Behavioral alterations in antibiotic-treated mice associated with gut microbiota dysbiosis: Insights from 16S rRNA and metabolomics. Front. Neurosci..

[145] Hammouda Z.K., Wasfi R., Abdeltawab N.F. (2023). Hormonal drugs: Influence on growth, biofilm formation, and adherence of selected gut microbiota. Front. Cell. Infect. Microbiol..

[146] Zhao H., Jiang X., Chu W. (2020). Shifts in the gut microbiota of mice in response to dexamethasone administration. Int. Microbiol..

[147] Barkus A., Baltrūnienė V., Baušienė J., Baltrūnas T., Barkienė L., Kazlauskaitė P., Baušys A. (2024). The gut-brain axis in opioid use disorder: Exploring the bidirectional influence of opioids and the gut microbiome-a comprehensive review. Life.

[148] Mlíchová J., Paluch Z., Šimandl O. (2023). Pain and analgesic related insomnia. Pain Manag. Nurs..

[149] Song B.C., Bai J. (2021). Microbiome-gut-brain axis in cancer treatment-related psychoneurological toxicities and symptoms: A systematic review. Support. Care Cancer.

[150] Zhong H., Jiang M., Yuan K., Sheng F., Xu X., Cui Y., Sun X., Tan W. (2025). Alterations in gut microbiota and metabolites contribute to postoperative sleep disturbances. Anim. Models Exp. Med..

[151] Sutanto C.N., Xia X., Heng C.W., Tan Y.S., Lee D.P.S., Fam J., Kim J.E. (2024). The impact of 5-hydroxytryptophan supplementation on sleep quality and gut microbiota composition in older adults: A randomized controlled trial. Clin. Nutr..

[152] Momeniyan Z., Dehghan P., Tutunchi H., Azadi H., Azizi-Soleiman F. (2025). The effect of date seed powder supplementation on anxiety- and depression-like behaviours, sleep quality and tryptophan-kynurenine metabolism in patients with type 2 diabetes: Targeting gut-brain axis. Br. J. Nutr..

[153] Tanihiro R., Yuki M., Sasai M., Haseda A., Kagami-Katsuyama H., Hirota T., Honma N., Nishihira J. (2023). Effects of prebiotic yeast mannan on gut health and sleep quality in healthy adults: A randomized, double-blind, placebo-controlled study. Nutrients.

[154] Thompson R.S., Gaffney M., Hopkins S., Kelley T., Gonzalez A., Bowers S.J., Vitaterna M.H., Turek F.W., Foxx C.L., Lowry C.A. (2021). Ruminiclostridium 5, parabacteroides distasonis, and bile acid profile are modulated by prebiotic diet and associate with facilitated sleep/clock realignment after chronic disruption of rhythms. Brain Behav. Immun..

[155] Liu Y., Chen Y., Zhang Q., Zhang Y., Xu F. (2025). A double blinded randomized placebo trial of Bifidobacterium animalis subsp. lactis BLa80 on sleep quality and gut microbiota in healthy adults. Sci. Rep..

[156] Lee H.J., Hong J.K., Kim J.K., Kim D.H., Jang S.W., Han S.W., Yoon I.Y. (2021). Effects of probiotic NVP-1704 on mental health and sleep in healthy adults: An 8-week randomized, double-blind, placebo-controlled trial. Nutrients.

[157] Li J., Zhao J., Ze X., Li L., Li Y., Zhou Z., Wu S., Jia W., Liu M., Li Y. (2024). Lacticaseibacillus paracasei 207-27 alters the microbiota-gut-brain axis to improve wearable device-measured sleep duration in healthy adults: A randomized, double-blind, placebo-controlled trial. Food Funct..

[158] Magzal F., Shochat T., Haimov I., Tamir S., Asraf K., Tuchner-Arieli M., Even C., Agmon M. (2022). Increased physical activity improves gut microbiota composition and reduces short-chain fatty acid concentrations in older adults with insomnia. Sci. Rep..

[159] Zheng T.S., Gao X.R., Gu C., Ru Y.N., Xu R.P., Yang Y.H., Wang D.H. (2025). Long-term exercise mitigates energy expenditure and inflammatory responses induced by sleep deprivation in mice. Biomolecules.

[160] Bian Z., Zhang W., Feng Z., Gu L., Qin P., Lu Y., Chen X., Hu M., Cai L., Su L. (2025). Ziziphi spinosae semen extract ameliorates insomnia by regulating hypothalamic gaba/glu balance and gut microbiota lactobacillus johnsonii. J. Funct. Foods.

[161] He H., Li M., Qiu Y., Wu Z., Wu L. (2024). Washed microbiota transplantation improves sleep quality in patients with sleep disorder by the gut-brain axis. Front. Neurosci..

[162] Baek J.S., Ma X., Park H.S., Lee D.Y., Kim D.H. (2025). Bifidobacterium longum p77 and lactiplantibacillus plantarum p72 and their mix-live or heat-treated-mitigate sleeplessness and depression in mice: Involvement of serotonergic and gabaergic systems. Cells.

[163] Fang H., Yao T., Li W., Pan N., Xu H., Zhao Q., Su Y., Xiong K., Wang J. (2023). Efficacy and safety of fecal microbiota transplantation for chronic insomnia in adults: A real world study. Front. Microbiol..

[164] Arab A., Lempesis I.G., Garaulet M., Scheer F.A.J.L. (2025). Sleep and the Mediterranean diet: A systematic review and meta-analysis. Sleep Med. Rev..

[165] Yaghtin Z., Beigrezaei S., Yuzbashian E., Ghayour-Mobarhan M., Khayyatzadeh S.S. (2022). A greater modified Mediterranean diet score is associated with lower insomnia score among adolescent girls: A cross-sectional study. BMC Nutr..

[166] Yang Y., Yu M., Lu Y., Gao C., Sun R., Zhang W., Nie Y., Bian X., Liu Z., Sun Q. (2024). Characterizing the rhythmic oscillations of gut bacterial and fungal communities and their rhythmic interactions in male cynomolgus monkeys. Microbiol. Spectr..

[167] Bajaj P., Sharma M. (2025). Chrononutrition and gut health: Exploring the relationship between meal timing and the gut microbiome. Curr. Nutr. Rep..

[168] Binks H., Vincent G.E., Gupta C., Irwin C., Khalesi S. (2020). Effects of diet on sleep: A narrative review. Nutrients.

[169] Hong M., Zhang R., Liu Y., Wu Z., Weng P. (2022). The interaction effect between tea polyphenols and intestinal microbiota: Role in ameliorating neurological diseases. J. Food Biochem..

[170] Pérez-Jiménez J., Agnant K., Lamuela-Raventós R.M., St-Onge M.P. (2023). Dietary polyphenols and sleep modulation: Current evidence and perspectives. Sleep Med. Rev..

[171] Zhang F., Fan D., Huang J., Zuo T. (2022). The gut microbiome: Linking dietary fiber to inflammatory diseases. Med. Microecol..

[172] Choe U. (2025). Role of dietary fiber and short-chain fatty acids in preventing neurodegenerative diseases through the gut-brain axis. J. Funct. Foods.

[173] Yoo S., Jung S.C., Kwak K., Kim J.S. (2024). The role of prebiotics in modulating gut microbiota: Implications for human health. Int. J. Mol. Sci..

[174] Bowers S.J., Summa K.C., Thompson R.S., González A., Vargas F., Olker C., Jiang P., Lowry C.A., Dorrestein P.C., Knight R. (2022). A prebiotic diet alters the fecal microbiome and improves sleep in response to sleep disruption in rats. Front. Neurosci..

[175] Kaur S., Sharma P., Mayer M.J., Neuert S., Narbad A., Kaur S. (2023). Beneficial effects of GABA-producing potential probiotic Limosilactobacillus fermentum L18 of human origin on intestinal permeability and human gut microbiota. Microb. Cell Factories.

[176] Rosas-Sánchez G.U., Germán-Ponciano L.J., Puga-Olguín A., Soto M.E.F., Medina A.Y.N., Muñoz-Carillo J.L., Rodríguez-Landa J.F., Soria-Fregozo C. (2025). Gut-brain axis in mood disorders: A narrative review of neurobiological insights and probiotic interventions. Biomedicines.

[177] Haarhuis J.E., Kardinaal A., Kortman G.A.M. (2022). Probiotics, prebiotics and postbiotics for better sleep quality: A narrative review. Benef. Microbes.

[178] Ahmad S.R., AlShahrani A.M., Kumari A. (2025). Effects of probiotic supplementation on depressive symptoms, sleep quality, and modulation of gut microbiota and inflammatory biomarkers: A randomized controlled trial. Brain Sci..

[179] Lozar Krivec J., Bratina P., Valcl A., Lozar Manfreda K., Petrovčič A., Benedik E., Obermajer T., Bogovič Matijašić B., Šetina U., Rupnik M. (2024). Effects of Limosilactobacillus reuteri DSM 17938 in neonates exposed to antibiotics: A randomised controlled trial. Benef. Microbes.

[180] Xie Y., Liu S., Chen X.J., Yu H.H., Yang Y., Wang W. (2021). Effects of exercise on sleep quality and insomnia in adults: A systematic review and meta-analysis of randomized controlled trials. Front. Psychiatry.

[181] Zhao B., Sun J., Xiang L., Su Z. (2025). Exercise as a modulator of gut microbiota for improvement of sleep quality: A narrative review. Front. Neurosci..

[182] Das M., Muralitharan G., Saini S., Patra S. (2025). Sleep, gut microbiota, and mind-body medicine. Brain Behav. Immun. Integr..

[183] Lau R.I., Su Q., Ching J.Y.L., Lui R.N., Chan T.T., Wong M.T.L., Lau L.H.S., Wing Y.K., Chan R.N.Y., Kwok H.Y.H. (2024). Fecal microbiota transplantation for sleep disturbance in post-acute COVID-19 syndrome. Clin. Gastroenterol. Hepatol..

[184] Wu C., Dou J., Song X., Yang F., Liu X., Song W., Zhang X. (2025). Gut microbiota: A new target for the prevention and treatment of insomnia using Chinese herbal medicines and their active components. Front. Pharmacol..

[185] Guan B., Liu X., Hu Z., Hu X., Liu S., Yang K., Zhou L., Yu L., Yang J., Chen S. (2025). Sleep-promoting mechanism of Lactobacillus plantarum P8 combined with traditional Chinese medicine revealed by network pharmacology, microbiome, metabolome, and transcriptome analyses. Food Biosci..

